# P-914. Comparison of Linezolid and Contezolid Exposure on Platelet Levels in ICU Patients: A Single-Center Retrospective Cohort Study

**DOI:** 10.1093/ofid/ofaf695.1120

**Published:** 2026-01-11

**Authors:** Yuxin Zuo, Jingyi Liang, Zhongqing Chen, Hongbing Hu, Zhenhua Zeng

**Affiliations:** Nanfang Hospital, Southern Medical University, Guangzhou, Guangdong, China (People's Republic); Nanfang Hospital, Southern Medical University, Guangzhou, Guangdong, China (People's Republic); Nanfang Hospital, Southern Medical University, Guangzhou, Guangdong, China (People's Republic); Nanfang Hospital, Southern Medical University, Guangzhou, Guangdong, China (People's Republic); Nanfang Hospital, Southern Medical University, Guangzhou, Guangdong, China (People's Republic)

## Abstract

**Background:**

Linezolid is often associated with the development of thrombocytopenia. Previous studies have demonstrated that contezolid, a novel oxazolidinone, exhibits favorable efficacy in treating infections caused by drug-resistant Gram-positive bacteria. This study aimed to compare the effects of contezolid and linezolid exposure on platelet levels in ICU patients.

Figure 1. Flow diagram of patient selection

**Figure 1.**
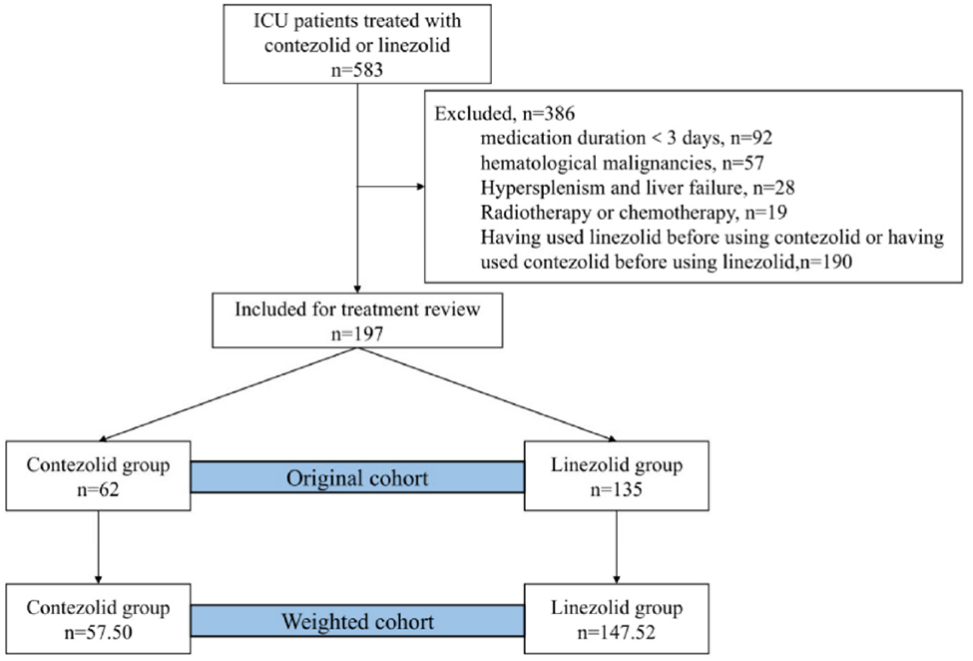
Flow diagram of patient selection

**Methods:**

This retrospective study included adult patients admitted to the Department of Critical Care Medicine at Nanfang Hospital, Southern Medical University (Guangzhou, China), from March 2023 to December 2024. Patients who received at least 3 days of contezolid or linezolid therapy were included. Logistic regression models were used to compare the incidence of thrombocytopenia, a reduction of greater than 30% from the baseline platelet count, between the two groups. To mitigate confounding bias in observational data, inverse probability of treatment weighting (IPTW) was applied to balance baseline characteristics, followed by outcome analysis.

**Results:**

A total of 197 patients met the inclusion criteria, with 62 receiving contezolid and 135 receiving linezolid. Significant differences were observed between the groups in age (67.6 vs. 57.5 years, *P* < 0.001) and baseline platelet levels (164.0 vs. 218.0, *P* = 0.037). The incidence of thrombocytopenia in the contezolid group was lower than that in the linezolid group (32.3% vs 54.8%). However, logistic regression analysis showed that the difference between the two groups did not reach statistical significance (OR 0.50, 95%CI 0.25-1.01, *P* = 0.058). After adjusting for confounding factors by IPTW, regression analysis showed that the risk of thrombocytopenia in the contezolid group was lower than that in the linezolid group, and the difference was statistically significant (OR 0.48, 95%CI 0.24-0.93, *P* = 0.034). Additionally, the contezolid group demonstrated a higher clinical efficacy rate than the linezolid group in the weighted cohort (84.5% vs. 59.9%, OR 3.41, 95% CI 1.5-8.58, *P* = 0.006).

**Conclusion:**

In ICU patients, contezolid exposure was associated with a lower incidence of thrombocytopenia and higher clinical efficacy compared to linezolid.

**Disclosures:**

All Authors: No reported disclosures

